# A practical guide to the implementation of AI in orthopaedic research part 8: Resource management checklist for AI‐driven research projects in orthopaedics

**DOI:** 10.1002/jeo2.70623

**Published:** 2026-01-31

**Authors:** Umile Giuseppe Longo, Benedetta Bandini, Maristella Saccomanno, Pieter D'Hooghe, Bálint Zsidai, Jacob F. Oeding, Felix Conrad Oettl, Kristian Samuelsson, Alessandro De Sire, Robert Feldt, Yinan Yu, ESSKA Artificial Intelligence Working Group

**Affiliations:** ^1^ Fondazione Policlinico Universitario Campus Bio‐Medico Roma Italy; ^2^ Research Unit of Orthopaedic and Trauma Surgery, Department of Medicine and Surgery Università Campus Bio‐ Medico di Roma Roma Italy; ^3^ Department of Bone and Joint Surgery Spedali Civili Brescia Italy; ^4^ Department of Medical and Surgical Specialties, Radiological Sciences, and Public Health University of Brescia Brescia Italy; ^5^ Aspetar Orthopedic and Sports Medicine Hospital, Aspire Zone Doha Qatar; ^6^ Sahlgrenska Sports Medicine Center Gothenburg Sweden; ^7^ Department of Orthopaedics, Institute of Clinical Sciences Sahlgrenska Academy University of Gothenburg Gothenburg Sweden; ^8^ Department of Orthopedics Skåne University Hospital Malmö/Lund Sweden; ^9^ Department of Orthopedic Surgery Balgrist University Hospital University of Zürich Zurich Switzerland; ^10^ Hospital for Special Surgery New York New York USA; ^11^ Department of Orthopaedics Sahlgrenska University Hospital Mölndal Sweden; ^12^ Department of Medical and Surgical Sciences University of Catanzaro “Magna Grecia” Catanzaro Italy; ^13^ Department of Computer Science and Engineering Chalmers University of Technology Gothenburg Sweden

**Keywords:** artificial intelligence, checklist, orthopaedics, project, resource management

## Abstract

Artificial intelligence (AI) is transforming a multitude of medical fields, including orthopaedic surgery. AI‐driven approaches such as machine learning, deep learning, natural language processing and large language models are being increasingly employed across various aspects of orthopaedic practice, offering innovative solutions for diagnostics, patient care and surgical training. To successfully execute an AI‐driven orthopaedic project, the initial step involves defining the aim and rationale of the project. The study must be designed to answer a clinically relevant topic in a way that influences the behavior of the health professional and leads to better patient outcomes. Once this planning phase is complete, selecting the most appropriate AI model becomes crucial, as models differ in applications, costs and required staff expertise. After model selection, successful AI implementation demands ongoing monitoring and adaptation to ensure optimal performance and reliability. Achieving the best and most ethical outcomes requires interdisciplinary collaboration, combining clinical expertise with technological proficiency. Ultimately, a comprehensive approach to AI integration can lead to transformative advancements in orthopaedic surgery and medical research, paving the way for improved patient care and innovative treatment solutions.

**Level of Evidence:**

Not applicable.

**Abbreviations**
AI: artificial intelligence
CPU: central processing unit
CST: collaborative science team
DL: deep learning
EFFORT: European Federation of National Associations of Orthopaedics and Traumatology
EMA: European Medicines Agency
FDA: Food and Drug Administration
FHIR: fast healthcare interoperability resources
GDPR: general data protection regulation
GPU: graphics processing unit
HER: electronic health record
HIPAA: Health Insurance Portability and Accountability Act
LIME: local interpretable model‐agnostic explanations
LLM: large language model
MI‐CLAIM: clinical artificial intelligence modeling
ML: machine learning
NLP: natural language processing
PROBAST‐AI: prediction model risk of bias assessment tool
SHAP: SHapley additive exPlanations
TRIPOD + AI: transparent reporting of a multivariable prediction model for Individual prognosis or diagnosis with artificial intelligence

## INTRODUCTION

Artificial Intelligence (AI) is a term first coined by McCarthy et al. in 1955, referring to the scientific objective of producing intelligent computers that could execute duties associated with human labor [4]. This discipline combines computer science and mathematics, with the original aim of matching, and eventually surpassing, human intellect using intelligent computer algorithms [14]. AI‐driven approaches such as machine learning (ML), deep learning (DL) and natural language processing (NLP) are being increasingly employed across various aspects of orthopaedic practice.

The application of AI has evolved from theoretical exploration to practical implementation, particularly within a diverse range of medical specialties, including orthopaedic surgery [25]. In fact, a scoping review on AI in orthopaedics in 2023 highlighted a considerable increase in AI‐related publications over the preceding years, highlighting this shift in clinical integration. Practical medical orthopaedic AI applications are plentiful, including imaging for fracture identification, tumor recognition, surgical planning decision‐making support, prediction of outcomes, reinjury risks, mortality rates and hospital stays, and utilizing digital twin technology to create personalized virtual patient models for improved treatment planning, postoperative care, rehabilitation monitoring and surgical training [12, 25].

Such implementations will establish the foundation for a personalized health management strategy, which is anticipated to reduce health care costs by lowering the rates of hospitalizations, outpatient consultations and therapeutic interventions [8, 22].

However, while AI has the potential to revolutionize patient care and operational efficiency, the successful implementation of AI‐driven projects requires careful planning and resource management. For researchers and clinicians approaching this rapidly evolving domain, understanding the key components that contribute to the success of an AI project is essential. These components include not only technological resources but also considerations related to data management, interdisciplinary collaboration and ethical guidelines.

Thus, the aim of this article is to provide a comprehensive resource management checklist for developing a successful AI‐driven orthopaedic research project, basing on the information provided in the previous articles of this learning series and current available checklists.

## CURRENT AVAILABLE CHECKLISTS

As the field advances, a growing number of AI models are being evaluated in interventional clinical trials. However, there is still a need for rules to properly inform readers and users about the application of the models in the projects. For this reason, the minimum information about clinical artificial intelligence modeling (MI‐CLAIM) and the transparent reporting of a multivariable prediction model for Individual Prognosis or diagnosis with artificial intelligence (TRIPOD + AI) guidelines were proposed, representing a step further towards transparency and utility of AI in health care. These guidelines serve two functions: first, to enable direct assessment of clinical effect, including fairness and bias; and second, to enable quick replication of the technical design process of a clinical AI study.

The MI‐CLAIM includes six topics that deserve consideration when assessing DL AI models, and they seek to influence clinical use of AI models. The domains assessed are study design, data partitioning for model training and testing, optimization and final model selection, performance evaluation, model inspection and reproducible pipeline [15].

TRIPOD + AI, on the other hand, describes a 27‐item checklist for AI prediction model research with checks concerning the title, abstract, introduction, methods, open science practices, patient and public involvement, results and discussion. The recommendations in TRIPOD + AI focus on the transparent reporting of research involving prediction models, rather than on the methods for developing or evaluating such models [3].

While TRIPOD + AI focuses on both traditional statistical and ML/AI methods, the MI‐CLAIM is specialized to DL techniques [19].

An adaptation of the TRIPOD framework designed for large language models (LLMs) is identified by the TRIPOD‐LLM. While TRIPOD‐AI extends the original TRIPOD guidelines to cover ML–based prediction models, TRIPOD‐LLM focuses specifically on models built using LLMs that process free‐text inputs. TRIPOD‐LLM covers domains such as context variability, prompt engineering, LLM configuration settings like temperature and interpretability of natural language outputs, all characteristics of LLM‐driven models [5].

In addition to these strategies, the FUTURE‐AI checklist offers a methodical framework for managing the development and application of reliable and ethical AI in the healthcare setting. It comprises six core principles: fairness, ensuring AI models work equitably across diverse populations; universality, promoting broad applicability across different clinical settings; Traceability, enabling transparency through clear documentation of data and model decisions; usability, ensuring AI tools are user‐friendly and integrate seamlessly into clinical workflows; robustness, guaranteeing performance stability across varying conditions; and explainability, making AI decisions interpretable for clinicians and patients [11, 16, 17].

Another available framework is the METRIC checklist, which developed five clusters that are crucial for ensuring reliable AI in healthcare: the measurement process concentrates on detecting data errors, such as device inaccuracies and human mistakes, in addition to evaluating the completeness and credibility of the data; timeliness ensures the relevancy of the data over time, by taking into account variables like age and currency; representativeness highlights the importance of balanced and varied data, making sure that all pertinent demographics and subpopulations are included; informativeness ensures the data is clear, meaningful and free of redundancy, while also taking into account the significance of missing values and important attributes; ultimately, consistency verifies that the dataset is uniform among subpopulations by guaranteeing that its formatting, logical structure and statistical characteristics are all the same.

Finally, the prediction model risk of bias assessment tool (PROBAST‐AI) checklist provides a comprehensive framework to appraise the risk of bias and the overall quality of already published AI‐based prediction models, emphasizing whether studies appropriately address issues such as overfitting, data leakage and performance assessment across different populations or settings. The aim of this tool is to promote more robust, transparent and generalizable AI models for clinical use by standardizing the assessment of study quality and bias [2].

By applying these principles, researchers and developers can enhance the openness, dependability and equity of AI‐driven healthcare solutions, ensuring that AI systems are built on accurate, unbiased and actionable data [21].

## RESOURCE MANAGEMENT

In this study, resource management refers to the strategic allocation and coordination of technological, data, human, financial and ethical resources to successfully implement an AI‐driven orthopaedic research project. It ensures that all necessary components, such as hardware, expertise and regulatory compliance, are effectively managed to achieve the goals of the project.

### Project planning

The first part of resource management in the context of an AI‐driven orthopaedic project should include defining the research aim and rationale. The study must be designed to answer a clinically relevant topic in a way that influences the behavior of the health professional and leads to better patient outcomes. Moreover, the intervention must be defined, scalable and relevant to the issue identified, and it must produce results that can be applied to similar clinical situations in a variety of demographics and disease prevalence [6].

The second challenge in developing AI‐driven research is assembling a representative, diverse data set to train the most appropriate model type chosen. It is essential to train a model with data that most closely resemble the exact format and quality of data expected during use.

For instance, for a ML model that is intended to be used at the point of care cannot be trained on patient data that would typically only be available in the electronic health records (EHRs) at later moments in time. When large enough data sets are available, modern models can be successfully trained to map noisy inputs to noisy outputs. Moreover, convergence of a common data format, such as fast healthcare interoperability resources (FHIR), can improve data aggregation, giving patients discretion over who gets access to their data for model development or running [20].

The third and final phase would focus on model creation, validation, deployment and review. During this phase, researchers choose and assess the best model for their AI‐powered study. Once model training is complete, and the model performs well on the originally selected test set, external validation is crucial to reduce the risk of inherent biases before it is deployed and, later, fine‐tuned for further performance improvements. Following deployment, researchers undertake a project review to evaluate outcomes and performance, allowing them to identify lessons learned and best practices for future initiatives [10].

### Resources—Equipment and materials

Ensuring the availability of appropriate resources to execute a project is essential. More specifically, it is necessary to identify the specific type of AI that is most suited to the needs of the project. As the field of AI continues to evolve, it becomes essential to grasp the distinct features and functions of different technologies to ensure optimal project results [9]. However, we also note that as the ML and AI fields are undergoing extensive evolution, a very brief overview is provided in this manuscript; while more details can be found in earlier papers in this learning series [17, 25, 26] orthopaedic researchers should consider teaming up with statistics and AI experts to get an up‐to‐date view of the latest and most suitable technology at the time of commencing the project.

For classical ML models, such as logistic regression, support vector machines or decision trees, no specialized hardware is typically required. These models are computationally lightweight and can be trained and deployed effectively on standard central processing units (CPUs). They are often used with structured tabular data, where large‐scale data pipelines are typically not involved. In such cases, the demands on storage and networking are minimal. However, for applications requiring real‐time predictions, such as patient monitoring or real‐time clinical decision support, low‐latency, high‐bandwidth networking becomes important to ensure timely data flow and responsiveness.

In contrast, DL applications, such as image classification, medical image segmentation or DL models for clinical text analysis, require significantly more designated computing power. Parallel processing infrastructure, such as graphics processing units (GPUs), is essential for training and inference, especially when dealing with high‐resolution images or large volumes of unstructured data. These tasks also require substantial storage to manage datasets, model checkpoints and logs. If privacy concerns prevent centralizing patient data, federated learning offers a decentralized approach by training models locally on each data source. This setup, however, adds complexity and requires sufficient edge hardware and secure, reliable networking infrastructure.

### Resources—Time

While the timelines of individual AI‐driven orthopaedic research projects vary based on the proposed goals of the project and the availability of the previously discussed resources, the major phases of the development process are highlighted below:

- Research objective and data description
  Focuses on defining the problem, the goals, and, importantly, analyzing and creating a detailed description of the available data. This stage sets the foundation for the project and helps ensure it aligns with medical as well as business goals. It's critical to analyze and describe not only the directly available data but to consider also additional, relevant data that might provide additional performance. Based on the data description and analysis of the expected need for cleaning, labeling and segmentation of the data that might be needed, a time estimate for the next phase, data collection, can be made. Ethical approvals might need to be sought in this phase, unless already achieved.
- Data collection and cleaning
  The data collection phase can vary wildly in time depending on to what extent the data is already available in digital form or has to be scanned, entered or accessed through other, nondigital means. Even when data is readily available, this phase can easily take weeks since data typically has to be cleaned due to missing data, variation in formats and units that have been used, etc. And in projects where manual labeling or segmentation of data is needed this phase can easily grow to consume a major part of the whole project and taking several months.
- Model creation and initial training
  Allows for initial performance estimates based on baseline models, helping guide further development.
- Model refinement
  Can often be the most extensive phase, taking months or more. During this time, the team iterates on the solution, refining the model as well as the dataset and its cleaning and use, based on results from each sprint to achieve a production‐ready state.
- Validation and regulatory approval
  To ensure that the projects meet safety, reliability and clinical effectiveness standards, thorough testing in various populations and clinical settings is necessary to confirm their accuracy and broader applicability. Regulatory agencies, like the European Medicines Agency and the U.S. Food and Drug Administration evaluate these technologies based on criteria such as patient safety, data transparency and clinical validity. This process is often complex and time‐consuming, requiring strict compliance with established guidelines that frequently change [18].
- Project improvement

It is a continuous process that begins after deployment. This phase involves ongoing monitoring and reviewing of the model to adapt to changing data and ensure consistent performance [10]. As the patient population, the medical condition itself, data collection procedures and other involved systems and staff changes, the model and the solution will also have to change. Secondary effects on the clinical setting where the model and solution is being used, as well as secondary effects on patients, will have to be continuously considered and actions taken to mitigate any negative effects.

### Resources—Finance

ML models can enhance physicians’ abilities to anticipate future events by analyzing the health trajectories of many patients and drawing on expertise beyond an individual experience of the practitioner. However, the data abstraction process can be labor‐intensive, necessitating strong validation and quality control measures [1]. As a result, budgets may need to account for these costs, which can vary depending on the scope and size of the clinical practice [20]. In fact, AI is linked with significant capital expenses and financial strain on healthcare systems, which may impede its wider implementation. Regardless, well‐planned cost‐benefit evaluations might reveal if its use in orthopaedics leads to cost‐effective therapies [12].

Moreover, particularly for small healthcare facilities and developing countries, which stand to profit the most as they may not have access to world‐class physicians, the cost of creating and implementing AI systems makes it challenging to use this technology. Accessibility to these cutting‐edge diagnostic and treatment options may be hampered by their high cost [7].

In addition, specific ML technologies can be relatively expensive and may not be affordable for all hospitals and clinics. Therefore, the choice of AI technology to employ in an AI‐driven research project must consider the available money, the unique requirements of the project, and the anticipated return on investment in terms of improved patient outcomes and operational efficiency. Cost‐effective alternatives and scalable solutions should also be considered to offer greater accessibility and viability for a wider variety of medical institutions [13].

**Figure 1:**
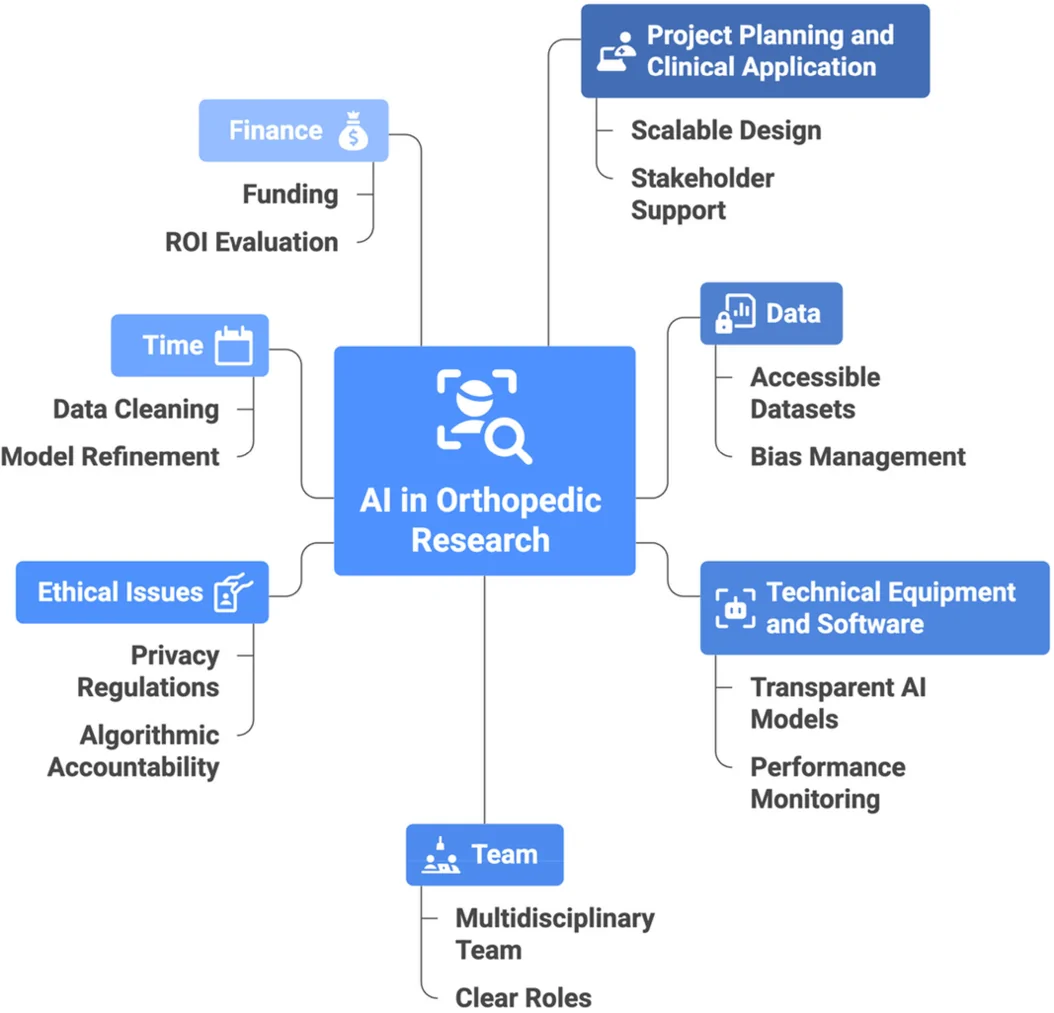
Key points in AI research in orthopedics. AI, Artificial Intelligence; ROI, return on investment.

On the other hand, a financial analysis should also consider the long‐term effects that a new AI model or technology can enable. The focus should not solely be on replacing small parts of currently manual procedures and steps. New technologies and models can also enable whole new ways of working that have more widespread effects and thus can both increase and decrease costs. We recommend a broad analysis is performed on the financial as well as medical effects that the introduction of the AI technology leads to both in the short, medium and long‐term. These analyses can be helped by review articles that have looked at the economic impact of AI in healthcare (Table 1, Figure 1) [24].

**Table 1:** Resource management checklist.

Category	Subcategory	Checklist item	Yes/no
Project planning and clinical application (3)	Research aim and relevance	Research question is clinically significant and addresses a real‐world orthopedic need	
	Intervention design	Intervention is clearly defined, scalable and applicable across settings and populations	
	Stakeholder involvement	Clinical and technical stakeholders are engaged from the beginning with a clear plan	
	Impact on clinical workflow	Project is designed to integrate into or inform clinical workflows	
	Outcome measures	Patient‐centered and clinical outcome metrics are clearly defined	
Data (20)	Data availability and access	Relevant data sources identified and accessible	
	Data format and standards	Data conforms to clinical standards (e.g., FHIR)	
	Data representativeness	Dataset includes diverse demographic and clinical variables	
	Bias and fairness assessment	Data reviewed for sampling bias and potential sources of algorithmic bias	
	Partitioning and labeling	Data partitioned for training, validation and testing with clear labeling strategy; the partition should closely resemble the exact format and quality of data expected during use	
Technical equipment and software	AI model selection	Appropriate type and model (ML, DL, NLP, LLM) based on clinical goal	
	Model development and validation	Model trained, validated and externally tested (use a checklist such as TRIPOD‐AI, TRIPOD‐LLM or PROBAST‐AI)	
	Explainability and transparency	Tools like SHAP/LIME used to enhance model interpretability	
	Integration and compatibility	Software infrastructure aligns with hospital systems/EHRs	
	Infrastructure and tools	In case of deep learning model development, access to computing resources (e.g., GPU); tools like TensorFlow, PyTorch available	
	Deployment and monitoring	Monitoring for model drift, performance decay and feedback loops established	
Team	Team composition	Collaborative Science Team (CST) selection done; project manager, data scientists, software engineers, IT support, orthopaedic surgeons and nurses and ethics committee assigned	
	Role definition	Clear responsibilities for each team member	
	Training and capacity building	Training provided on data handling, clinical context or AI methodology as needed	
Ethical issues	Patient privacy and consent	Data protection complies with GDPR/HIPAA; informed consent protocols in place	
	Algorithmic accountability	Responsibility chain for AI decisions clearly documented	
	Ethical evaluation	Project reviewed by ethics committee; ethical evaluation following the European Federation of National Associations of Orthopaedics and Traumatology (EFORT) guidelines	
	Transparency to patients	Ability to explain AI‐driven decisions to patients and clinicians	
Time (12)	Planning duration	Time allocated for stakeholder consultation and data access planning	
	Research objective and data description	8–10 weeks	
	Data collection and cleaning	High variance	
	Model creation and validation duration	6–8 weeks planned for initial model development and performance validation	
	Model refinement and iteration duration	4 months reserved for iterative development and improvement cycles	
	Postdeployment monitoring	Ongoing time committed to postdeployment monitoring and updates	
Finance	Budget planning	Total project cost estimated, including labor, tech, compliance and training	
	Capital investment evaluation	Cost‐benefit analysis performed (clinical ROI, operational efficiency gains)	
	Scalability and sustainability	Options for low‐cost tools, scalability to broader clinical settings considered	
	Funding secured	Internal or external funding identified and allocated	

Abbreviations: AI, artificial intelligence; CST, collaborative science team; DL, deep learning; EFFORT, European Federation of National Associations of Orthopaedics and Traumatology; EGR, electronic health record; FHIR, fast healthcare interoperability resources; GDPR, general data protection regulation; GPU, graphics processing unit; HIPAA, Health Insurance Portability and Accountability Act; LLM, large language model; ML, machine learning; NLP, natural language processing; PROBAST‐AI, Prediction model risk of bias assessment tool; ROI, return on investment; TRIPOD + AI, transparent reporting of a multivariable prediction model for individual prognosis or diagnosis with artificial intelligence; TRIPOD + LLM, transparent reporting of a multivariable prediction model for individual prognosis or diagnosis with large language models.

## CONCLUSIONS

AI has the potential to support orthopaedic research and clinical practice by enhancing diagnostics, treatment planning and patient care. Successful integration of AI technologies in orthopaedics requires a strategic approach to resource management, including selection of appropriate AI models, assembly of representative data sets and evaluation of the performance of the tool, and collaboration among multidisciplinary teams. Ethical considerations surrounding data privacy, informed consent and accountability must be prioritized to ensure the responsible use of AI in healthcare.

While current frameworks in literature, such as MI‐CLAIM, TRIPOD + AI, TRIPOD‐LLM, PROBAST‐AI, FUTURE‐AI and METRIC establish essential guidance for AI transparency, validity and ethical issues, this final article of this learning series expands these topics by addressing the pragmatics of resource utilization.

Finally, by addressing these challenges, AI continues to drive significant improvements in orthopaedic outcomes, contributing to ongoing cost savings and enhancing patient experiences.

## AUTHOR CONTRIBUTIONS

**Umile Giuseppe Longo**: Conceptualization; validation; writing—original draft preparation; writing—review and editing; project administration. **Benedetta Bandini**: Methodology; investigation; writing—original draft preparation. **Maristella Saccomanno**: Formal analysis; data curation. **Pieter D'Hooghe**: Formal analysis; data curation. **Bálint Zsidai**: Conceptualization; validation; investigation; writing—review and editing; supervision; project administration. **Jacob F. Oeding**: Methodology; investigation. **Felix Conrad Oettl**: Conceptualization; validation; writing—review and editing; supervision; project administration. **Kristian Samuelsson**: Conceptualization; validation; writing—review and editing; visualization; project administration. **Alessandro De Sire**: methodology; formal analysis; data curation. **Robert Feldt**: Conceptualization; validation; writing—review and editing; visualization; project administration. **Yinan Yu**: Methodology; writing—review and editing. All authors have read and agreed to the published version of the manuscript.

### ESSKA Artificial Intelligence Working Group

This manuscript is part of a series of publications by the ESSKA Artificial Intelligence Working Group. Related previous publications include: Zsidai et al. [25, 26], Oettl et al. [17], Winkler et al. [23].

## CONFLICT OF INTEREST STATEMENT

TKS is the member of the Board of Directors of Getinge AB (publ) and medtech advisor to Carl Bennet AB.

## ETHICS STATEMENT

The authors have nothing to report.

## Data Availability

Data sharing is not applicable to this article as no datasets were generated or analysed during the current study.
